# Employing the Precautionary Principle to Evaluate the Use of E-Cigarettes

**DOI:** 10.3389/fpubh.2016.00005

**Published:** 2016-02-04

**Authors:** Ashley M. Bush, James W. Holsinger, Lawrence D. Prybil

**Affiliations:** ^1^Department of Health Management and Policy, University of Kentucky, Lexington, KY, USA; ^2^Department of Preventive Medicine and Environmental Health, University of Kentucky, Lexington, KY, USA

**Keywords:** precautionary principle, e-cigarettes, tobacco, smoking, public health

## Abstract

Electronic cigarettes (e-cigarettes) have emerged onto the public market as an alternative to tobacco cigarettes; however, science is inconclusive as e-cigarettes have not been thoroughly investigated, including their short- and long-term risks and benefits (1, 2). The question arises of whether e-cigarettes will become the future tobacco crisis. This paper connects the precautionary principle to the use of e-cigarettes in an effort to guide decision-makers in the prevention of adverse health outcomes and societal costs.

## Using the Precautionary Principle to Evaluate the Use of E-Cigarettes

Electronic cigarettes (e-cigarettes) are marketed as a smoking cessation tool, and their use is increasing particularly among middle and high school students in the US, but among adults as well (3, 4). Despite the high prevalence of use, e-cigarettes have not been thoroughly investigated (5); the benefits and risks of their use are unknown, including the chemicals being consumed (1). Voluntary consumer and health-care reports cite hospitalization for pneumonia, seizures, disorientation, congestive heart failure, and hypotension as e-cigarette related (5). However, studies so far show mixed evidence, including no significant relationship regarding smoking cessation and e-cigarettes (6, 7) and potential for cessation (8, 9), while others suggest e-cigarettes encourage “dual use” in conjunction with smoking tobacco (3, 10, 11). The adoption of e-cigarettes is strongly promoted; e-cigarettes are advertised on radio and television, and in print as occurred in the 1950s with tobacco (3).

Although other ethical principles are available, this paper utilizes the precautionary principle (PP)^1^ in considering the issue of the use of e-cigarettes. This principle may be utilized in an effort to increase protection and to minimize risk from harmful activities, such as e-cigarette use, in an effort to prevent another nicotine crisis. The PP is used to guide decision-making when science is inconclusive and forces individuals to promote “the greatest good for the greatest number” (16). The PP encourages planning, precaution, and prevention rather than a reaction to harmful activities. In recent years, the PP has been cited in national legal codes and international treaties, as well as having been utilized by commercial organizations to describe potential harm from products (14, 17–19).

## Consideration of Cigarette Use as a Failed Public Health Policy

Tobacco production and consumption have resulted in what Fink defines as a crisis (20). A crisis has four stages: (1) prodromal, (2) acute, (3) chronic, and (4) resolution (20). As early as the 1950s, warning signs (prodromes) were present regarding the use of tobacco (20) as scientific studies of smoking and its adverse health effects were being published (21), prompting a US government response in an effort to prevent the impending crisis. “Smoking and Health,” a report by the Surgeon General’s Advisory Committee, indicated the harmful effects of smoking. The Report quotes US Surgeon General Leroy E. Burney, who stated in 1957 that “excessive smoking is one of the causative factors of lung cancer.” The Report referred to Burney’s 1959 article in the Journal of American Medical Association, which stated that “smoking is a principal factor in the increased incidence of lung cancer” and increases lung cancer risks. Meanwhile, tobacco companies’ industry-sponsored research examining the effects of smoking on laboratory animals was inconclusive (21). Some commentators have thought that the tobacco industry manipulated or withheld results to maximize earnings (22–24).

Despite these warnings and research, the production and use of smoking tobacco continued, with the tobacco industry refining its traditional marketing practices. It began offering filtered, low-tar and nicotine options, coupons, and sponsorships (e.g., concerts, sporting events, and other promotions) to increase cigarette consumption. Almost two decades passed following the first studies before product labeling was required, and radio and television advertising were banned to help decrease overall cigarette consumption (25, 26).

Following the warning signs of the first crisis, the tobacco industry worked to control communication – a key to good crisis management (20). Their efforts were visible in their marketing of tobacco products, which helped to thwart legal action against the industry. Tobacco companies developed a Tobacco Industry Research Committee to combat claims regarding adverse outcomes from tobacco use (22, 27). Some hypothesize that the warning signs were the reason that the tobacco industry invested in the international market and various varieties of cigarettes (26).

The acute phase evolved – “the point of no return” (20) – after the tobacco companies’ 1994 Congressional testimony. At this time, the tobacco industry’s communication began to unravel as the CEOs of seven largest tobacco companies publicly stated that cigarettes were not addictive, statements contrary to a former tobacco company board member’s 1963 claim that “Nicotine is addictive. We are, then in the business of selling nicotine, an addictive drug” (28). This resulted in “misunderstanding and [the] erosion of trust” among the tobacco companies’ constituents (29). Prior to this time, the tobacco companies groomed their positive public image and gained the trust of consumers, as well as increased their profitability. This brief phase led to further investigation by the various parties involved, with the resulting transition to the chronic crisis phase.

In the chronic phase, public health agencies, government entities, and tobacco companies all tried to control the tobacco crisis through litigation. After the CEOs’ testimonies, information regarding the actual long-term effects of smoking was made available. Tobacco was determined to be the leading cause of preventable death in the US. In this situation, the efforts made to control the repercussions of the tobacco crisis were carried out by public health agencies, the various states, and individuals by holding the tobacco companies accountable for their product – cigarettes. In 1998, these efforts led to the $206 billion Master Settlement Agreement (30).

The tobacco crisis is currently in the chronic phase, such as beginning January 1, 2016, Hawaii will become the first US state to raise the legal purchasing age of cigarettes to 21 years (31). In actuality, the Institute of Medicine reports that raising the national minimal legal age to 21 could help prevent 223,000 premature deaths, 50,000 deaths from lung cancers, and over 4 million years of life lost for persons born from 2000 to 2019 (1). Medical costs continue to rise as tobacco-related diseases, and mortality rates remain high with the overall mortality three times greater for smokers than those who have never smoked (32). Thus, tobacco-related diseases are still the most preventable cause of death in the US (32). Smoking-related costs were $289 billion from 2009 to 2012, of which $133 billion provided direct medical care and $156 billion was based on smoking-related lost productivity (32). Although the tobacco industry spent $8 billion in cigarette marketing in 2011 (32), the use of tobacco is still a winnable public health battle (32).

## A Bridge Between a Past and New Public Health Problem

The tobacco crisis exemplifies the need for improved policies to address smoking, a past public health problem, and e-cigarettes, a new public health problem. The PP provides an effective approach, utilizing an upstream methodology to reduce harm to humans and the environment. The principle was developed in the 1930s (15), and it is derived from the German word *Vorsorgeprinzip*, which means forward looking (12). Prior use of the principle included the creation of legislation regarding water pollution, natural resource exploitation, and toxic substance use (12, 19, 33). The PP states,

When an activity raises threats of harm to human health or the environment, precautionary measures should be taken even if some cause and effect relationships are not fully established scientifically. In this context the proponent of an activity, rather than the public, should bear the burden of proof. The process of applying the Precautionary Principle must be open, informed and democratic and must include potentially affected parties. It must also involve an examination of the full range of alternatives, including no action (Wingspread Statement on the Precautionary Principle, 1998) (14).

Moreover, the PP originated as a link between “uncertain scientific information and a political responsibility … [in order] to prevent damage to human health” (13).

### Advantages of the Precautionary Principle

The PP has been both praised and criticized. Proponents praise it because it protects individuals, who may require policy efforts to control exposure and limit risk due to their vulnerabilities and/or inability to change exposures. It calls for persons to use common sense when science is uncertain or absent (i.e., if a product appears to be negatively affecting the environment or individuals, use should diminish or cease while alternatives are explored). Scientific evidence does not always advance quickly enough to establish absolute cause and effect due to uncertainty (i.e., it takes time to understand the long-term effects of tobacco use). Acknowledging this, the PP suggests that actions should be undertaken to prevent further harm to an increasing number of individuals during this period of uncertainty.

**Figure 1:**
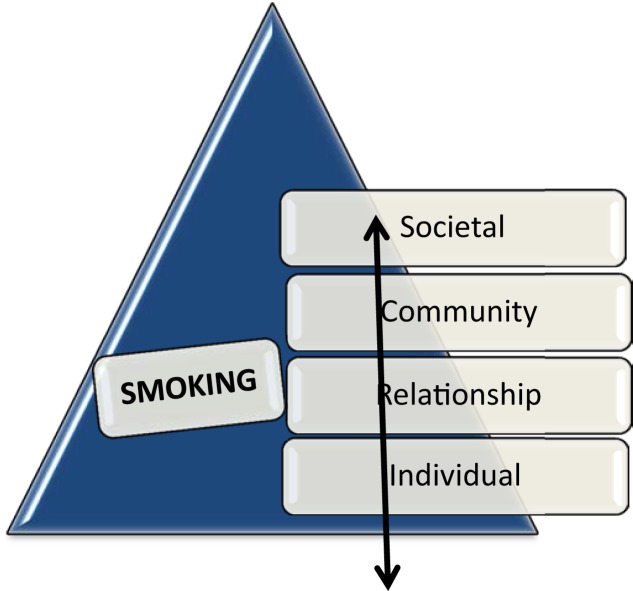
**Sociological model**. Adapted from the Framework for Prevention and Protection (34).

The PP calls for an examination of the activity of interest using a socioecological perspective (34), where individuals, industry, and policy-makers work together to understand the problem [e.g., Socioecological Model – Figure 1 (34)]. It calls for industries to wait to introduce products to society until they are able to demonstrate minimal risk. Also, the PP appeals for a more educated populace, resulting in informed stakeholders who are then able to exercise autonomy in risk-taking. Citizens desire the ability to choose, even if their choices are irrational. By promoting open and democratic decision-making, “group think” is limited (35). Different perspectives are considered, which can lead scientists, policy-makers, and the public to think outside of the norm. Finally, the PP spurs the quest for safer alternatives that may expand the use sustainable and reusable products in order to reduce harm.

### Criticisms of the Precautionary Principle

Critics state that the PP has economic consequences in that it limits industrial production and time and causes the loss of jobs, thus creating financial hardship for workers and their families. Precautions may result in presumptions concerning an activity’s effect and stigmatize the activity prior to scientific studies being undertaken (i.e., premature conclusions). Some investigators suggest that “false-positives” will occur and distract focus and resources away from the actual burden of the undertaking (36). Critics also argue that the PP is too conservative as it encourages bans on products when only slight exposure and/or harm exist.

Discussion and debate have centered on the lack of a universal operational definition of the PP, which may complicate when and how the principle is exercised (37). Detractors also state that the PP suppresses innovation and technology (38), and scientists argue that it encourages decision-making without scientific support (38). Because the PP pushes science inquiry, when feasible, for justification in the use of certain products or activities, a misuse or misunderstanding of the science by policy-makers and industry may occur. Even though the PP calls for all parties to be informed, scientists and industry leaders may sanction products as safe based on their findings but may not reveal study results in their entirety.

## Application of the Precautionary Principle to E-Cigarettes to Address an Impending Public Health Crisis

The tobacco crisis justifies the current application of the PP to e-cigarette use. E-cigarettes are devices that deliver nicotine to the body with vaporized delivery mechanisms that were introduced to the US market in 2004. Currently, although e-cigarettes are regulated by the Food and Drug Administration (FDA), research on risks and benefits of their use is scant. The FDA found that some e-cigarettes contain known carcinogens (e.g., anabasine, nitrosamines, diethylene glycol, etc.) (2). However, the FDA stated that conclusions cannot be drawn due to product variability (2). Research has revealed 466 different e-cigarette brands in the US (39). In fact, US researchers examined one e-cigarette brand and found nanoparticles, silicate beads, and metals (lead, nickel, and chromium) in the aerosol vapor and cartomizer fluid (40). These metals have a well-documented history of causing lung (e.g., impaired function, cancer, respiratory irritation, and pulmonary fibrosis), nervous system, and kidney damage when inhaled and/or digested (40).

Implementation of the PP may prevent deleterious health effects in the future through research and regulation of these untested devices. A looming concern is that nicotine, fruit flavorings, and other e-cigarette additives may encourage teenagers and children to initiate use of tobacco cigarettes (41). The American Academy of Pediatrics (2013) stated that children may decide to use e-cigarettes because they are perceived to be safer than conventional cigarettes (41). The CDC found that 1.8 million middle and high school students have tried e-cigarettes (42). The age of smoking initiation is basic to the argument that smoking is a “pediatric disease” (43). Additionally, adolescent e-cigarettes users were more likely over the next year to smoke tobacco than non-users (11). Some states are exercising the PP through proactive legislation by prohibiting e-cigarette sales to minors (e.g., Kentucky) (44), raising the minimum age limits for purchasing e-cigarettes to 19 (e.g., Alaska) and 21 (e.g., New York City) (1), and amending existing smoking bans to include e-cigarettes (e.g., New Jersey) (44). Nonetheless, e-cigarettes may be purchased in other states and from online retailers without age restrictions; thus, permitting children and teenagers to obtain e-cigarettes and initiate their use.

Ethically speaking, the PP encourages accountability, transparency, and responsibility from the e-cigarette industry for their products. The PP urges policy-makers to move away from a policy based on presumption of innocence until proven guilty for activities and products to one that is based on the finding of guilty until proven innocent. Should e-cigarettes be regarded as dangerous until proven safe? Should the e-cigarette industry be required to prove that its products are free of risk to humans?

## A Retrospective Application of the Precautionary Principle to the Tobacco Crisis

Scientific inquiry early established the consequences of smoking tobacco; however, American society allowed politics and the tobacco companies to dictate action in this instance. The tobacco industry failed to accept the scientific evidence – studies showing a causal relationship between smoking and cancer. Questionable decision-making by all parties allowed the crisis warning signs to intensify with time, as did the ill health effects of cigarette use.

Although introduced to the US in 1998, the PP, relying on scientific evidence, may have helped to alleviate or prevent the impact of the tobacco crisis. As early as 1950, scientific research was initiated to understand the effects of tobacco use. On the other hand, tobacco manufacturers countered these scientific efforts by claiming that its own research demonstrated no causal relationship between smoking and health. The first scientific studies in conjunction with the Surgeon General Reports should have resulted in “bright spots” for the government and public as the health risks of tobacco consumption were demonstrated (45). Public health agencies acted on the reports by requiring cigarette labeling and television-advertising bans, but despite these efforts tobacco sales increased in the mid-1970s (25). Utilizing the PP may have guided leaders to examine alternatives that would result in minimizing harm for the greatest good and that would expedite the process of protecting the public. However, even today, over 3,200 children initiate smoking cigarettes daily (32). Policy-makers were puzzled as to how to regulate cigarettes since they were considered neither food nor drug and the effects of smoking could not be observed until decades later. For this reason, utilizing the PP could have proved useful as further understanding of the effects of tobacco use would have been required, potentially preventing progression into the acute crisis stage.

The PP results in a search for alternative products or practices to achieve similar benefits from cigarette use (e.g., stress reduction). The PP could have discouraged the use of harmful chemicals in cigarettes, and encouraged development of other means of nicotine delivery. Currently, the Patient-Centered Outcomes Research Institute (PCORI) is conducting comparative research on the effectiveness of smoking cessation compared to long-term nicotine replacement therapy for high-risk individuals. Study findings may provide evidence to support nicotine alternatives for conventional smoking (46). Perhaps, e-cigarettes or safer alternatives may have been developed earlier as an alternative to conventional tobacco smoking. Bernheim et al. note that public perception regarding the use tobacco changed with the introduction of nicotine replacement therapy, which further echoes the benefits of the implementation of the PP (43). Also, its use may have resulted in a call for removal of cigarettes from the marketplace when multiple ill effects were observed through the Surgeon General Reports, health care, and individual lawsuits against the tobacco industry, which were prevalent as early as 1954 (21, 22).

The PP helps link “uncertain scientific information” to “political responsibility” in an effort to prevent poor health outcomes (13). The public should not be required to bear the proof of the negative effects of tobacco use; the tobacco industry should bear the proof. Moreover, the use of the PP calls for open and democratic decision-making for all stakeholders (12). Social responsibility may result in reduced political and tobacco industry domination of the issue, as well as more public and stakeholder participation to prevent further tobacco-related risks. Putatively, if the PP had been applied in regards to the uncertain risk of tobacco smoking, smoking may have been banned entirely, lives saved, tobacco-related diseases prevented, and health-care costs reduced.

## Shifting from Past Policy to Future Policy

In conclusion, the PP should be applied to e-cigarettes in order to understand the potential benefits and/or long-term consequences of e-cigarette use since currently the scientific research data are inconclusive and regulation virtually non-existent. Use of the PP as a tool will benefit public health policy-makers as they consider the inherent political and ethical dilemmas concerning population health related to e-cigarette use by examining evidence on safer alternatives.^2^ At the state and local levels, existing smoking bans can be amended to include the prohibition of e-cigarettes in public places, and minimum age limits for purchasing e-cigarettes established or raised. On the national level, agencies should prioritize e-cigarette research, regulate advertising and marketing practices, call for premarket regulation, as well as require product labeling in order to educate consumers concerning the potential health risks of e-cigarette use. A proactive and preventive rather than a reactive approach is required to address human activities resulting in health risks. Policy-makers must speak for those without voices, consumers must hold manufacturers ethically accountable, and public health leaders must demand health equity for all.

## Author Contributions

AB: contributed by content development of the perspectives paper along with substantial writing and editing of the paper. JH and LP: provided substantial input to the content of the paper and reviewed and edited it prior to submission.

## Conflict of Interest Statement

The authors declare that the research was conducted in the absence of any commercial or financial relationships that could be construed as a potential conflict of interest.

## Footnotes

^1^Raffensperger and Tickner (12); Martuzzi and Tickner (13); Wingspread Statement on the Precautionary Principle (14); O’Riordan and Cameron (15). Authoritative sources. Subsequent sections of this paper will discuss its application to electronic cigarettes and America’s health.

^2^The following recommendations are not necessarily those of the authors, nor are they exhaustive or comprehensive of all the potential means of applying the Precautionary Principle to address the impending electronic cigarette crisis.
